# Retention of an Enormous Quantity of Urine

**Published:** 1859-01

**Authors:** 


RETENTION OF AN ENORMOUS QUANTITY OF URINE.—We have received from Dr. E. B. P. Kelly, one of the resident-physicians in Blockley Almshouse, the following notes, interesting on account of their showing the great extent to which the urinary bladder can be dilated:—

"A. H., aged forty, born in Ireland, was admitted into the hospital on the 25th of June, 1858, laboring under a severe form of tertiary syphilis, which had made fearful ravages on the bones of the skull and on the inferior extremities. He had also several organic strictures of the urethra, the result of repeated attacks of gonorrhœa, one, which was very firm and resisting, being situated near the extremity of the organ, another, of less extent and more dilatable, at the triangular ligament, and, a third, between those two points, which was easily overcome. The patient succeeded in concealing his true condition until 11 P.M. of the same date, when he was suddenly taken ill and became very much alarmed. I was sent for, and upon my arrival found him in great agony, with hurried breathing, quick fluttering pulse, and anxious countenance. The state of the patient led me, at once, to inquire into the condition of the urinary bladder, and, to my utter astonishment, he informed me that he had not passed water for five days and a half. An examination of the abdomen, which was greatly distended, the bladder having risen two inches above the umbilicus, confirmed, in great measure, the statements of the patient.

"I found it impossible to introduce a silver catheter, on account of the resistance of the strictures, and ordered a warm bath and some relaxing medicine, after which I succeeded by gentle and persistent efforts in introducing a small bougie. Allowing this to remain for a few minutes, I used a second and then a third instrument of larger size, when the catheter passed with considerable facility, and was followed by the escape of the enormous quantity of one hundred and thirty-two ounces of urine.

"The patient ultimately recovered under the use of the iodide of potassium and bichloride of mercury, conjoined with tonics and a good diet."

